# High tidal volume mechanical ventilation-induced lung injury in rats is greater after acid instillation than after sepsis-induced acute lung injury, but does not increase systemic inflammation: an experimental study

**DOI:** 10.1186/1471-2253-11-26

**Published:** 2011-12-28

**Authors:** Jan Willem Kuiper, Frans B Plötz, AB Johan Groeneveld, Jack J Haitsma, Serge Jothy, Rosanna Vaschetto, Haibo Zhang, Arthur S Slutsky

**Affiliations:** 1 From the Department of Paediatric Intensive Care, VU University Medical Centre, Amsterdam, The Netherlands; 2 Department of Anaesthesia and Interdepartmental Division of Critical Care Medicine, The Keenan Research Centre at the Li Ka Shing Knowledge Institute of St. Michael's Hospital; 3 Department of Paediatrics, Tergooiziekenhuizen, Blaricum, The Netherlands; 4 Intensive Care, VU University Medical Centre, Amsterdam, The Netherlands; 5 Department of Laboratory Medicine, University of Toronto, Toronto, Ontario, Canada; 6 Departments of Anaesthesiology and Critical Care Medicine, University of Eastern Piedmont, Novara, Italy

**Keywords:** Ventilator-induced lung injury, acute lung injury, sepsis, cytokines, lung histology, mechanical ventilation.

## Abstract

**Background:**

To examine whether acute lung injury from direct and indirect origins differ in susceptibility to ventilator-induced lung injury (VILI) and resultant systemic inflammatory responses.

**Methods:**

Rats were challenged by acid instillation or 24 h of sepsis induced by cecal ligation and puncture, followed by mechanical ventilation (MV) with either a low tidal volume (Vt) of 6 mL/kg and 5 cm H_2_O positive end-expiratory pressure (PEEP; LVt acid, LVt sepsis) or with a high Vt of 15 mL/kg and no PEEP (HVt acid, HVt sepsis). Rats sacrificed immediately after acid instillation and non-ventilated septic animals served as controls. Hemodynamic and respiratory variables were monitored. After 4 h, lung wet to dry (W/D) weight ratios, histological lung injury and plasma mediator concentrations were measured.

**Results:**

Oxygenation and lung compliance decreased after acid instillation as compared to sepsis. Additionally, W/D weight ratios and histological lung injury scores increased after acid instillation as compared to sepsis. MV increased W/D weight ratio and lung injury score, however this effect was mainly attributable to HVt ventilation after acid instillation. Similarly, effects of HVt on oxygenation were only observed after acid instillation. HVt during sepsis did not further affect oxygenation, compliance, W/D weight ratio or lung injury score. Plasma interleukin-6 and tumour necrosis factor-α concentrations were increased after acid instillation as compared to sepsis, but plasma intercellular adhesion molecule-1 concentration increased during sepsis only. In contrast to lung injury parameters, no additional effects of HVt MV after acid instillation on plasma mediator concentrations were observed.

**Conclusions:**

During MV more severe lung injury develops after acid instillation as compared to sepsis. HVt causes VILI after acid instillation, but not during sepsis. However, this differential effect was not observed in the systemic release of mediators.

## Background

Acute lung injury and its most severe manifestation, the acute respiratory distress syndrome (ARDS), result from direct or indirect pulmonary insults. A direct, primary or pulmonary insult directly affects the lung parenchyma. In contrast, an indirect, secondary or extrapulmonary insult follows from an acute systemic inflammatory response [1]. In a clinical study, involving patients suffering from pneumonia, peritonitis or polytrauma, these types of injury differed in terms of respiratory mechanics and response to positive end-expiratory pressure [2]. Animal experiments showed additional differences in the pulmonary inflammatory response, ultra-structural characteristics, apoptosis and respiratory mechanics [3-6]. The distinction between these insults may have therapeutic implications regarding ventilator settings [7-9]. For example, predominant consolidation of the lung in direct injury as opposed to oedema and alveolar collapse in indirect injury might imply the potential for greater ventilator-lung induced injury (VILI) in the former since alveolar recruitability would be less and overdistension greater for a given tidal volume [2,10,11]. However, this concept is highly controversial [12-14]. One of the consequences of VILI is an increase in translocation of mediators involved in lung inflammation into the systemic circulation, thereby potentially contributing to remote organ injury and failure [15-20]. It is unknown, however, whether direct vs. indirect injuries differ in this respect, but greater propensity for VILI in the former could be associated with a greater systemic inflammatory response.

We hypothesize that the harmful effects of different ventilation strategies depend on the type of underlying lung injury. In this study we therefore set out to examine the effects of different ventilatory strategies on lung injury and systemic mediator release in direct and indirect pulmonary injury in rats. Direct pulmonary injury was induced by acid aspiration, regarded as an important risk factor for the development of ARDS [21-24]. Indirect pulmonary injury was generated by cecal ligation and perforation, a widely used animal model of sepsis - one of the main risk factors for ARDS [21]. We measured plasma mediators involved in inflammation, chemotaxis, cell adhesion and fibrinolysis that relate to remote organ dysfunction and outcome of acute lung injury [15-18,25-31].

## Methods

### Animal preparation

Animals were treated according to the Canadian national guidelines and with approval of the Animal Care Committee of St Michaels Hospital. Male Sprague Dawley rats (Charles Rivers, St Constan, QC, Canada) weighing 290-320 g were anesthetized with xylazine (Bayer, Toronto, ON, Canada) 10 mg/kg and ketamine (Bimeda-MTC, Cambridge, ON, Canada) 100 mg/kg given intraperitoneally. Anaesthesia was maintained by intravenous xylazine 1 mg/kg/h, ketamine 20 mg/kg/h; muscle relaxation was achieved by intravenous pancuronium bromide (Sabex Inc, QC, Canada) 0.6 mg/kg/h. During surgical procedures and ventilation, rats were supine on a heating pad and body temperature was maintained at 37°C. For blood sampling, fluid infusion and arterial blood pressure measurements, catheters were inserted into the right carotid artery and tail vein before stabilization. The arterial catheter was connected to a pressure transducer for continuous measurement of arterial blood pressure. During mechanical ventilation (MV) all animals received a continuous infusion of normal saline to maintain mean arterial blood pressure > 60 mmHg, and for patency of intravenous lines.

A pilot study was undertaken to establish the acid instillation protocol. Briefly, after anaesthesia and tracheotomy, a 14 G canula was inserted into the trachea and connected to a ventilator (Servo 300, Siemens, Munich, Germany); set to deliver a tidal volume (Vt) of 6 mL/kg and a positive end-expiratory pressure (PEEP) of 5 cm H_2_O. One animal was ventilated per ventilator per experiment. Arterial and venous catheters were inserted and hydrochloric acid (HCl, pH 2.0), 1 ml/kg, was rapidly instilled intratracheally at baseline using an aerosolizer (PennCentury Inc, Philadelphia, PA, USA). Instillation was followed by a recruitment manoeuvre (increase in PEEP to 25 cm H_2_O for 5 breaths). Rats were subsequently stabilized for 10 minutes and then randomized. Acid aspiration control rats received acid instillation alone after which they were sacrificed after the recruitment manoeuvre. Rats did not survive acid instillation without subsequent MV due to technical and ethical limitations; inclusion of time-matched controls was therefore impossible. The mortality rate of acid instillation was 13% before randomisation.

Sepsis was induced as follows. With the animal spontaneously breathing 40% oxygen, a laparotomy through a midline incision using an aseptic technique was performed. The coecum was ligated just below the ileocecal valve with 3-0 silk ligature, so that intestinal continuity was maintained. Using a 14-Gauge needle, the coecum was perforated in two locations, 1 cm apart, on the antimesenteric surface of the coecum, and the coecum was gently compressed until faeces were extruded. The bowel was then returned to the abdomen and the incision was closed using 4-0 silk ligature for both the muscle layer and skin. Subsequently, rats received 30 mL/kg 0.9% saline in the scruff of the neck and buprenorphine 30 μg/kg subcutaneously (Schering-Plough, Hertfordshire, UK). The rats breathed 40% oxygen until recovery from anaesthesia, and then were placed back in a cage with free access to food and water. Eight hours after surgery, rats received a 30 mL/kg 0.9% saline bolus i.p. Mortality rate of the model prior to randomisation was 6%. Twenty-four hrs after the induction of sepsis, rats were anaesthetized and tracheotomy was performed, with a canula (14 gauge) inserted into the trachea. Rats were connected to a ventilator; arterial and venous catheters were inserted followed by a 10-minute stabilization period with ventilation using Vt 6 mL/kg and PEEP 5 cm H_2_O.

### Experimental protocol

After stabilization, rats were randomly allocated to one of 4 groups: MV with either a low Vt (6 mL/kg) and PEEP 5 cm H_2_O (n = 10 per group) (LVt acid and LVt sepsis) or a high Vt (15 mL/kg), no PEEP (n = 10 per group) (HVt acid and HVt sepsis). Eight rats were immediately sacrificed after acid instillation (acid). Ten rats served as non-ventilated septic controls (sepsis) and were sacrificed 28 hr after induction of sepsis. Tidal volumes were selected that have shown to cause lung injury or proven to be non-injurious in rats [15-17], and inspiratory/expiratory ratio was 1:2. Normocapnia was maintained by adjusting respiratory rate. The fraction of inspired oxygen was set at 0.4 and increased when necessary in ventilated groups. At the end of the experiment a blood sample was taken and animals were sacrificed with an overdose of ketamine/xylazine.

Rats were ventilated for 4 hrs during which blood pressure and heart rate were measured continuously. Arterial blood samples were taken 30 min after randomization and every hour for blood gas analysis (Ciba Corning Model 248 blood gas analyser, Corning Medical, Medfield, MA, USA). For each blood sample a volume of maximum 100 μl was necessary. An equal amount of normal saline was administered intra-venously to compensate for the fluid loss. Total respiratory dynamic compliance (ml/cm H_2_O) was calculated by Vt/(Pplat-PEEP), where Vt is the applied tidal volume and Pplat is the plateau pressure measured every 30 minutes during an inspiratory pause on the ventilator. Interleukin-6 (IL-6), tumour necrosis factor-α (TNF-α) and macrophage inflammatory protein-2 (MIP-2) were measured in plasma by enzyme linked immunosorbent assay (ELISA) (Biosource, Camarillo, CA, USA). Lower detection limits for these assays are 8 pg/mL, 4 pg/mL and 1 pg/mL for IL-6, TNF-α and MIP-2 respectively. Soluble intercellular adhesion molecule-1 (sICAM-1) (R&D Systems Inc, Minneapolis, MN, USA) and active plasminogen activator inhibitor-1 (aPAI-1) (Innovative Research Inc, Southfield, MI, USA), lower detection limit 0.05 ng/mL), were measured in plasma with an ELISA, according to manufacturer guidelines.

After sacrifice, lungs were harvested and the left upper lobe was formalin fixed until further histological analysis. The left lower lobe of the lung was harvested, weighed, and dried overnight in a heated stove. Lung wet to dry (W/D) weight ratio was calculated by dividing wet by the dry lung weight. All left upper lung lobes were transversely cut immediately above the main brochus insertion and random sections, including posterior and anterior parts of the lungs, were assessed by a pathologist blinded to the experimental origin of the specimens. A quantitative morphometric analysis of alveolar collapse, perivascular haemorrhage, alveolar haemorrhage, perivascular oedema, vascular congestion, alveolar polymorphonuclear leukocytes, alveolar oedema and macrophages was performed, as has been described previously [32]. Each item was scored 0-3 (0 = normal; 1 = mild; 2 = moderate; 3 = severe) and then calculated for a total score of lung injury (scores potentially ranging from 0-24) [32].

### Statistical analysis

Data are expressed as mean ± standard error of the mean. When data were not normally distributed according to a Kolmogorov-Smirnov test (p > 0.05), data were ranked before analysis. The effects of MV in each model were tested using univariate analysis of variance and longitudinal data were compared using generalized estimating equations designed for the analysis of repeated measurements. Post hoc testing was performed according to Bonferroni. Using these tests the effects of the model were analysed for each parameter and these comparisons are described by acid instillation or sepsis. Subsequently the effects of MV were analysed and finally the model-dependent effects were analysed by determining the interaction between model and MV for each parameter. A value of P < 0.05 was considered statistically significant, we report exact p-values unless P < 0.001. All analyses were performed using SPSS 17.0 statistical software (SPSS Inc., Chicago, IL, USA).

## Results

### Hemodynamics and arterial blood gas analysis

Mean arterial pressure (MAP) did not differ between the two models at baseline. MV strategy did not influence MAP at baseline, but, as analyzed by the interaction between model and MV, the LVt sepsis group had a lower MAP compared to the HVt acid group at baseline. During the experiment, the sepsis groups had a lower MAP compared to the acid groups (P < 0.001). No effects of MV strategy or interaction were observed. After 4 h of MV, however, no effects of model or MV were observed. Analyses of the interaction showed that the MAP in the HVt sepsis group was lower than that in the LVt acid group (P = 0.01) (Table 1). Heart rate showed a similar pattern, although at baseline animals in the sepsis model had higher heart rates (P = 0.001). Towards the end of the experiment, heart rate did not differ among groups (Table 1). No differences in pH and P_a_CO_2 _were observed between the models and MV. More importantly, values remained within normal range.

**Table 1:** Hemodynamics and arterial blood gas analysis.

		Baseline	T = 0 hr	T = 2 hr	T = 4 hr
Mean arterial pressure (mmHg)	LVt acid^#^	81 ± 4.0	84 ± 3.9	91 ± 3.8	77 ± 2.6*
	HVt acid^#^	77 ± 3.4	88 ± 5.5	87 ± 2.7	72 ± 2.3
	LVt sepsis	75 ± 1.7	74 ± 2.6	68 ± 2.2	69 ± 2.5
	HVt sepsis	74 ± 2.2	80 ± 2.2	66 ± 2.1	65 ± 0.9
Heart rate (bpm)	LVt acid	269 ± 10	267 ± 11	332 ± 10	336 ± 12
	HVt acid	289 ± 8	249 ± 14	328 ± 7	345 ± 11
	LVt sepsis	322 ± 9^$^	322 ± 9	346 ± 15	353 ± 8
	HVt sepsis	350 ± 14^$^	360 ± 12	361 ± 10	350 ± 9
pH	LVt acid	7.41 ± 0.02	7.39 ± 0.01	7.37 ± 0.01	7.35 ± 0.01
	HVt acid	7.41 ± 0.02	7.38 ± 0.01	7.40 ± 0.01	7.30 ± 0.02
	LVt sepsis	7.44 ± 0.01	7.42 ± 0.01	7.38 ± 0.02	7.33 ± 0.02
	HVt sepsis	7.44 ± 0.01	7.44 ± 0.01	7.41 ± 0.02	7.38 ± 0.02
P_a_CO_2 _(mmHg)	LVt acid	39.4 ± 1.1	42.9 ± 1.8	42.4 ± 1.4	42.2 ± 1.4
	HVt acid	40.0 ± 1.2	43.8 ± 2.3	40.3 ± 1.3	45.8 ± 2.1
	LVt sepsis	37.1 ± 0.8	38.7 ± 1.78	38.0 ± 1.0	42.2 ± 1.0
	HVt sepsis	37.0 ± 1.5	38.7 ± 1.6	36.0 ± 0.7	36.0 ± 0.6

Mean arterial pressure (MAP) and heart rate in rats during 4 h of mechanical ventilation after acid instillation or during sepsis with low tidal volume (LVt) (6 mL/kg), with a positive end-expiratory pressure (PEEP) of 5 cm H_2_O or high tidal volume (HVt) (15 mL/kg) without PEEP. Data represent mean ± SEM. ^# ^p < 0.001 for model compared to sepsis during the experiment, * p = 0.01 compared to HVt sepsis, ^$ ^p = 0.001 for model compared to acid only at baseline.

### Effects on the lungs

**Figure 1:**
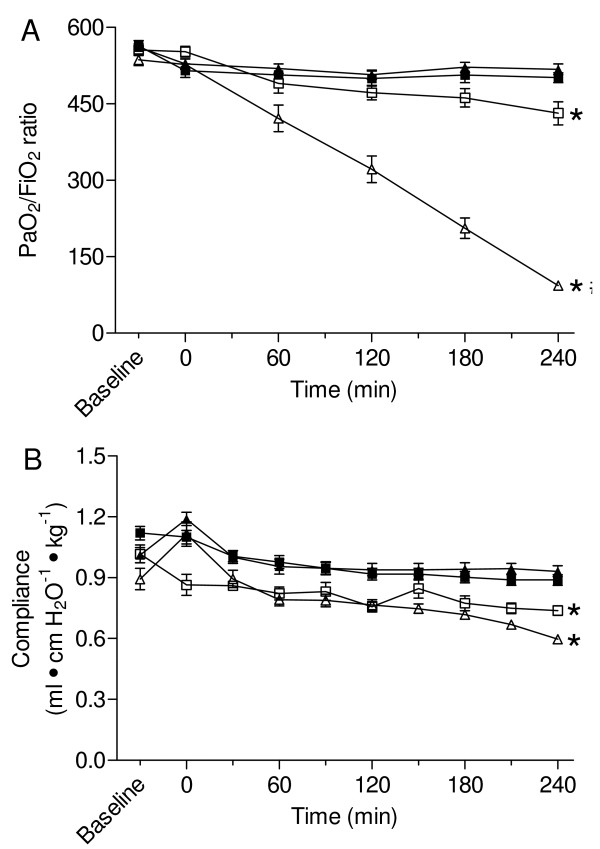
**P_a_O_2_/F_I_O_2 _ratio and lung compliance**. The P_a_O_2_/F_I_O_2 _ratio and compliance in rats ventilated for 4 h after acid instillation or during sepsis with low tidal volume (LVt) (6 mL/kg), with a positive end-expiratory pressure (PEEP) of 5 cm H_2_O or high tidal volume (HVt) (15 mL/kg), without PEEP. (A) P_a_O_2_/F_I_O_2 _ratio decreased after acid instillation irrespective of ventilation strategy. Mechanical ventilation (MV) after acid instillation decreased the P_a_O_2_/F_I_O_2 _ratio in contrast to HVt MV during sepsis. (B) Acid instillation decreased lung compliance as opposed to sepsis. No effects of MV were observed. * p < 0.001 for model compared to LVt and HVt sepsis, # p < 0.001 for interaction between model and MV. Open squares: LVt acid, open triangles: HVt acid, solid squares: LVt sepsis and solid triangles: HVt sepsis.

The P_a_O_2_/F_I_O_2 _ratio was similar among models at baseline and at randomisation (Figure 1A). The P_a_O_2_/F_I_O_2 _ratio was lower after acid instillation than during sepsis (P < 0.001). HVt affected the P_a_O_2_/F_I_O_2 _ratio (p < 0.001), however, as indicated by an interaction between MV and model (p < 0.001), HVt decreased P_a_O_2_/F_I_O_2 _ratio after acid instillation, but not during sepsis (Figure 1A). At baseline no differences in compliance were observed between the models (Figure 1B). The compliance was lower after acid instillation than during sepsis (P < 0.001) (Figure 1B). There was no effect of HVt on compliance in both models (P = 0.68), nor were there model dependent effects of HVt, as indicated by the absence of interaction between model and MV (P = 0.40).

### The W/D weight ratio and lung histology

**Figure 2:**
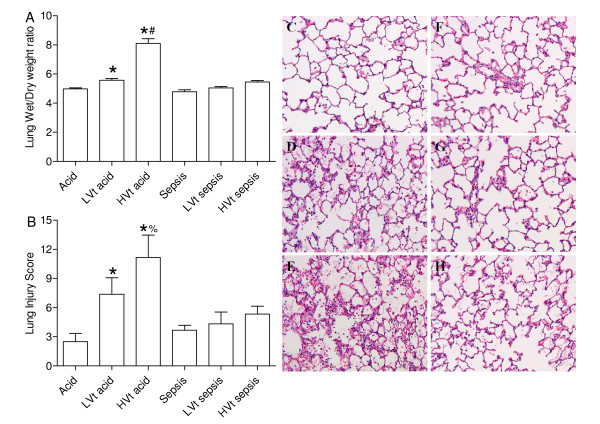
**Lung injury**. Wet to dry lung weight (W/D) ratios and lung injury scores in rats ventilated for 4 h after acid instillation or during sepsis with low tidal volume (LVt) (6 mL/kg), with a positive end-expiratory pressure (PEEP) of 5 cm H_2_O or high tidal volume (HVt) (15 mL/kg), without PEEP. Acid instillation increased W/D ratio as compared to sepsis. The effect of mechanical ventilation (MV) with HVt on W/D ratio was greater after acid instillation than during sepsis (A). Acid instillation increased lung injury score as compared to sepsis. The effect of HVt MV on lung injury score was greater after acid instillation than during sepsis. Panels (C) to (H) are representative micrographs demonstrating lung injury for: (C) Control rats, (D) and (E) demonstrate increased lung injury observed after acid instillation and MV with (D) LVt and (E) HVt. Sepsis alone increased lung injury (F); MV did not have an effect with either (G) LVt, or (H) HVt. Original magnification × 100. * p < 0.001 for model compared to LVt and HVt sepsis. # p < 0.001 for interaction between model and MV, % p = 0.04 for interaction between model and MV.

The lung W/D weight ratio was higher after acid instillation than during sepsis (P < 0.001). HVt increased the W/D weight ratio (P < 0.001), however, as indicated by an interaction between MV and model (P < 0.001), HVt increased W/D weight ratio after acid instillation, but not during sepsis (Figure 2A). The lung injury score was higher after acid instillation than during sepsis (P < 0.001). HVt increased the lung injury score (P < 0.001), however, as indicated by an interaction between MV and model (P = 0.04), HVt increased lung injury score after acid instillation, but not during sepsis (Figure 2B).

### Mediators

Acid instillation increased IL-6 plasma concentrations as compared to sepsis (P = 0.002). No effects of HVt on IL-6 plasma concentrations were observed, nor were there model-dependent effects of HVt (Table 2), therefore no significant differences were observed in IL-6 levels between LVt and HVt after acid instillation. Acid instillation increased TNF-α plasma concentrations as compared to sepsis (P = 0.005). HVt had no effects on plasma TNF-α concentrations. Effects of HVt did not depend on the model, i.e. no significant interaction between model and MV was observed (Table 2). Neither the model nor HVt had significant effects on plasma MIP-2 concentration (Table 2) and, additionally, there were no model dependent effects of HVt. Plasma ICAM-1 concentrations were higher during sepsis than after acid instillation (P = 0.031). MV with HVt did not increased plasma ICAM-1 concentrations as compared LVt, nor were there model dependent effects of HVt (Table 2). Neither the model nor MV had significant effects on plasma PAI-1 concentration (Table 2) and, additionally, there were no model dependent effects of HVt.

**Table 2:** Plasma concentrations of mediators.

	Acid	LVt acid	HVt acid	Sepsis	LVt sepsis	HVt sepsis
IL-6 (pg/ml)	321 ± 13	1113 ± 387^#^	758 ± 209^#^	312 ± 8	643 ± 190	393 ± 31
TNF-α (pg/ml)	39 ± 3	61 ± 9*	62 ± 12*	38 ± 2	42 ± 4	43 ± 10
MIP-2 (pg/ml)	4.6 ± 0.1	5.4 ± 0.4	5.2 ± 0.03	4.7 ± 0.3	5.0 ± 0.3	6.0 ± 0.8
sICAM-1 (ng/ml)	4.0 ± 0.3	30.9 ± 2.8^$^	22.6 ± 2.8^$^	31.4 ± 6.0	41.2 ± 6.7	32.2 ± 2.4
aPAI-1 (ng/ml)	0.0 ± 0.0	20.8 ± 2.1	12.5 ± 5.7	9.8 ± 3.0	18.5 ± 2.7	22.5 ± 3.1

Plasma concentrations of interleukin-6 (IL-6), tumour necrosis factor-α (TNF-α), macrophage inflammatory protein-2 (MIP-2), soluble intercellular adhesion molecule-1 (sICAM-1) and active plasminogen activator inhibitor-1 (aPAI-1) in rats ventilated for 4 h after acid instillation or during sepsis with low tidal volume (LVt) (6 mL/kg), with a positive end-expiratory pressure (PEEP) of 5 cm H_2_O or high tidal volume (HVt) (15 mL/kg) without PEEP. HVt after acid instillation did not affect plasma mediator levels. Acid instillation increased IL-6 and TNF-α plasma concentrations as compared to sepsis. Levels of sICAM-1 were lower in the acid group. Data represent mean ± SEM. ^# ^p=0.002 for model compared to LVt and HVt sepsis, ^* ^p=0.005 for model compared to LVt and HVt sepsis, ^$ ^p=0.031 for model compared to LVt and HVt sepsis.

## Discussion

The major finding of this study is that VILI develops only after HVt in combination with prior pulmonary acid instillation as shown by decreased P_a_O_2_/F_I_O_2 _ratio, increased W/D weight ratio and histological lung injury score. In contrast however, plasma mediator concentrations did not reflect this difference between the effects of HVt MV.

In this study we set out to examine the effects of different ventilatory strategies on lung injury and systemic mediator release in direct and indirect pulmonary injury in rats. This first warrants further explanation of the experimental models we used in this study. It is well known that both MV following acid instillation and MV during sepsis cause different degrees of acute lung injury assessed by histological examination [33]. Following intratracheal instillation of acid an acute inflammatory response in the lung develops within minutes, and a delayed injury usually develops 18 h after cecal ligation and puncture [17,33]. The intratracheal acid instillation model primarily targets the alveolar epithelium (direct injury) and can be used in conjuction with MV to further reproduce clinically relevant scenarios [22,33]. In humans, aspiration of gastric contents involves more than hydrochloric acid alone and the narrow difference between injurious and non-injurious acid concentrations is a potential disadvantage [33]. We therefore did a pilot study to determine the appropriate acid concentration. CLP-induced polymicrobial sepsis is one of the best and most widely used animal models for sepsis and organ injury, including lung injury, that mainly targets the endothelium (indirect hit) [33]. However, this model requires surgery and the bacterial inoculum is unknown [33]. Second, the two ventilation strategies we used in this study may require further explanation. For LVt ventilation we used 6 ml/kg with a PEEP of 5 cm H_2_O, a strategy that is used frequently and generally considered to be relatively non-injurious [16,32,34]. We compared LVt ventilation to a strategy that is generally considered to be injurious. Both the increased tidal volume of 15 ml/kg and the absence of PEEP contribute to the injurious nature of this strategy [15]. According to the literature, a Vt exceeding 15 ml/kg in the absence of PEEP would likely increase premature death rate [15]. In contrast, during sepsis, when lungs are not hit directly, for lung injury to occur, a Vt of more than 15 ml/kg may be needed to further injure the lungs [17]. However, recent reports have shown that a Vt of only 8 ml/kg may be injurious in previously healthy mice [30,35]. To balance between injury and mortality we therefore used an injurious strategy using a Vt of 15 ml/kg without PEEP.

The results of our study indicate that at the onset of randomization to HVt or LVt both groups had normal P_a_O_2_/F_I_O_2 _ratios and compliance, suggesting that there was little or no acute lung injury at that time. Although we did not perform a histological analysis at randomization it is likely that some degree of subclinical lung injury at randomization may have been present that was not reflected by oxygenation criteria. In line with this are the histological changes that were observed in the sepsis control group. As we hypothesized, the effects of the same HVt ventilatory strategy differed depending on the type of underlying pulmonary injury. Exposing lungs to 4 h of HVt MV revealed that profound VILI only occurred in the acid instillation group as compared to HVt MV during sepis. This was exemplified by a decreased P_a_O_2_/F_I_O_2 _ratio and compliance and increased histological lung injury score and W/D weight ratio. In contrast, LVt after acid instillation was not associated with VILI. During sepsis however, HVt ventilation did not decrease P_a_O_2_/F_I_O_2 _ratio and compliance, nor did it increase lung W/D weight ratio and lung injury score as compared to LVt MV during sepsis.

The results of the present study thus indicate that acid instillation and MV with a HVt, but not sepsis and HVt, synergistically lead to severe VILI. Acid instillation and sepsis, thus, prime the lungs differently and render lungs exposed to the former type of injury more susceptible to HVt VILI. This may be explained by the fact that in direct lung injury pulmonary alveolar filling by oedema, fibrin, collagen neutrophilic aggregates and/or blood leading to consolidation predominates [2,10-12]. Therefore it is likely that HVt ventilation after acid instillation, with a tidal volume distributed over a smaller aerated lung volume, led to repeated alveolar overdistension and subsequent aggravation of lung injury. In contrast, interstitial oedema and collapse may predominate in sepsis and HVt did therefore not further injure the lungs. This is supported by the increased lung injury score after HVt and acid instillation compared to sepsis, of which most items, such as alveolar haemorrhage and inflammatory cell infiltration, are associated with pulmonary consolidation. In line with this concept are observations made by Herrera et al. [17]. They observed lung injury only when tidal volumes of at least 20 ml/kg were used after CLP and they found mainly perivascular oedema and only moderate acute inflammatory infiltrates [17]. Others, however, have shown VILI during sepsis, but these studies applied much higher tidal volumes, ranging from 20-35 ml/kg, than we did [36-38]. Similarly, Altemeier et al, showed that MV with a tidal volume of 15 ml/kg after systemic lipopolysaccharide (LPS) did not cause gross histological changes [25]. In contrast, Chiumello et al. showed severe lung injury and premature death after MV with16 ml/kg without PEEP following acid instillation [15].

The second objective of our study was to to examine the effects of different ventilatory strategies on systemic mediator release. The systemic inflammatory response, in contrast to the pulmonary inflammatory response after different MV strategies following indirect and direct lung injury has been studied scarcely. For example, in a mouse model of direct (intra-tracheal endotoxin) and indirect (intra-peritoneal endotoxin) lung injury, Menezes et al. observed higher pulmonary IL-6, TNF-α mRNA and cellularity in bronchoalveolar lavage fluid in the former. They did not analyze blood levels, nor the effect of MV strategy [4]. Although Haitsma et al. administered LPS intratracheally and intraperitoneally to rats and showed that different PEEP levels (0 or 10 cm H_2_O) affected systemic TNF-α levels differently, they did not study other important mediators of systemic inflammation and only used high peak inspiratory pressures of 45 cm H_2_O [28]. Therefore, the differential effects of more clinically relevant MV strategies on decompartmentalization, i.e. the transfer of mediators from the intrapulmonary compartment to the systemic circulation, remain largely unknown. In our study, plasma mediator levels only partly reflected the differences in effects of ventilatory strategy on lung injury. The direct pulmonary insult (acid instillation) was associated with a greater increase in plasma IL-6 and TNF-α concentrations, but the indirect pulmonary insult (sepsis) resulted in increased plasma ICAM-1 concentrations. In contrast to parameters of lung injury however, HVt ventilation after acid instillation did not have an additional effect on mediator levels. Thus, in contrast to our hypothesis, MV induced systemic mediator release did not depend on the underlying type of lung injury. MV after acid instillation has previously shown to increase pro-inflammatory mediators such as IL-6 and TNF-α during MV [15]. The effects of MV on systemic mediator release during sepsis are less clear. For example, Herrera et al. showed that HVt ventilation during sepsis increased plasma TNF-α levels compared to LVt with PEEP but not to sepsis alone. In contrast, plasma IL-6 levels were increased after HVt ventilation compared to sepsis alone but not to LVt with PEEP [17]. Previously, we showed in healthy rats that different ventilation strategies affected lung histology without altering systemically circulating IL-6 levels [39] and similar results have been found by others [25]. After acid instillation, LVt MV may be sufficient for decompartmentalization to occur or HVt MV induced VILI was not severe enough since our levels of plasma TNF-α are lower after HVt MV than previously reported [15]. In sepsis HVt MV may not have been severe enough as reported previously by Herrera et al [17]. The systemic cytokine release during MV has been a topic of debate [40], differences in models, type and severity of lung injury and ventilatory settings may have led to different decompartmentalization in our study as compared to others [15,17,28]. Also, during sepsis the choice of mediators further complicates the debate, since different mediators have different patterns of excretion [41].

Our study has several limitations. We set out to test the hypothesis that harmful effects of different ventilation strategies depend on the type of underlying lung injury. Since most ICU patients requiring MV have underlying lung injury, we compared the effects of different MV strategies during indirect and direct lung injury. We therefore did not include healthy ventilated controls, time-matched non-ventilated sepsis controls were included. However, due ethical and technical limitations we were unable to include a time-matched control group for the acid instillation group. One may argue therefore that differences in severity of the first insult may account for the differential effects of HVt after acid instillation and during sepsis. However, histological analysis of lung injury directly after acid instillation showed only a marginally increased lung injury score. Prior to MV there were no indications that there was a difference in severity of the insults since oxygenation and lung compliance were comparable among the groups. Additionally, the different pathophysiologic mechanisms between direct and indirect lung injury make comparison of the severity of the initial insult in terms of lung injury precarious. Furthermore, the sole effect of MV on mediator levels in acid aspiration could not be determined. We did not make longitudinal measurements, so cannot completely exclude an interaction between type of pulmonary injury and MV on the course of plasma mediators. Gattinoni et al, observed different responses to PEEP between direct and indirect lung injury in patients previously [2]. We compared two ventilation strategies frequently used to study the effects of VILI; but did not further evaluate the role of PEEP.

## Conclusions

In conclusion, rats subjected to ventilation with HVt are more susceptible to VILI after pulmonary priming with acid instillation than after sepsis-induced lung injury. However, this difference in response was not observed in the systemic release of mediators. Therefore, the adverse effects of MV can differ between direct and indirect lung injury, and our data support even more cautious ventilator management in the former. Additionally, systemic mediator levels are not indicative for the severity of VILI. Further evaluation of the interaction between aetiology of acute lung injury, MV and systemic release of pulmonary inflammatory mediators in patients seems warranted.

## Key messages

• Lungs are more susceptible to ventilator induced injury after acid instillation than during sepsis

• The adverse effects of mechanical ventilation can differ between lungs exposed to direct or indirect lung injury

• Systemic mediator levels are not indicative for the severity of ventilator induced lung injury

## List of abbreviations

aPAI: active plasminogen activator inhibitor; ARDS: acute respiratory distress syndrome; ELISA: enzyme-linked immuno sorbent assay; HVt: high tidal volume; IL: interleukin; LPS: lipopolysaccharide; LVt: low tidal volume; MAP: mean arterial pressure; MIP: macrophage inflammatory protein; MV: mechanical ventilation; PEEP: positive end-expiratory pressure; sICAM: soluble intercellular adhesion molecule; TNF: tumor necrosis factor; VILI: ventilator-induced lung injury; Vt: tidal volume; W/D: wet to dry weight.

## Competing interests

The authors declare that they have no competing interests.

## Authors' contributions

JWK, FBP, ABG and AS conceived the study, planned the overall experimental design and wrote the manuscript. JWK and JH carried out the experiments; SJ's input was critical for the section on histology and advised on morphogical studies. RV and HZ advised in the experimental design. All authors have read and approved the final manuscript.

## Pre-publication history

The pre-publication history for this paper can be accessed here:

http://www.biomedcentral.com/1471-2253/11/26/prepub
